# Robust encoding of scene anticipation during human spatial navigation

**DOI:** 10.1038/srep37599

**Published:** 2016-11-22

**Authors:** Yumi Shikauchi, Shin Ishii

**Affiliations:** 1 Graduate School of Informatics, Kyoto University, Kyoto 606-8501, Japan; 2 ATR Cognitive Mechanisms Laboratories, Kyoto 619-0288, Japan

## Abstract

In a familiar city, people can recall scene views (e.g., a particular street corner scene) they could encounter again in the future. Complex objects with multiple features are represented by multiple neural units (channels) in the brain, but when anticipating a scene view, the kind of feature that is assigned to a specific channel is unknown. Here, we studied neural encoding of scene view anticipation during spatial navigation, using a novel data-driven analysis to evaluate encoding channels. Our encoding models, based on functional magnetic resonance imaging (fMRI) activity, provided channel error correction via redundant channel assignments that reflected the navigation environment. We also found that our encoding models strongly reflected brain activity in the inferior parietal gyrus and precuneus, and that details of future scenes were locally represented in the superior prefrontal gyrus and temporal pole. Furthermore, a decoder associated with the encoding models accurately predicted future scene views in both passive and active navigation. These results suggest that the human brain uses scene anticipation, mediated especially by parietal and medial prefrontal cortical areas, as a robust and effective navigation processing.

During spatial navigation, one needs to identify forthcoming scene views to make decisions appropriately. How is such anticipation represented in the brain? One hypothesis is that the brain utilises one specific microcircuit for each particular scene view (*whole-scene model*), similar to the conventional idea of grandmother cells. Another hypothesis suggests that a single scene view, which is intrinsically multi-dimensional, is divided into a number of basic features. Each microcircuit is in charge of representing one of these features; for example, a basic feature could be that the forward direction is either blocked by a wall or passable. Many other hypotheses suggest the representation falls somewhere between these versions; for example, scene views could be divided into several groups (e.g., scene views with an abundance of walls could be grouped together), so that each microcircuit is in charge of one group and behaves differently for that group than for the others.

When a person views a complex object which has multiple features, multiple neural units are activated simultaneously^1^. However, it remains to be determined what feature is assigned to a given individual unit. In the present study, we aimed to examine neural representations of scene-view anticipation during performance of virtual three-dimensional (3D) navigation games by humans in a magnetic resonance imaging (MRI) scanner. Participants performed two types of spatial navigation tasks: a scene choice task and a motion decision task. In the former, we examined whether participants could predict the upcoming scene view accurately during passive viewing of navigationally relevant stimuli^2^. In the latter, we examined correlations between participants’ scene prediction accompanied by decision-making (action selection), and their brain activities at times when participants actively navigated toward destinations.

To explore the method of neural representation (defined as encoding hereafter) in the brain during scene anticipation, we applied novel data-driven analyses to the brain activities recorded by functional MRI (fMRI) during the scene choice task to model the encoding method. Our encoding models were then verified by their complementary decoding analyses. In fact, fMRI-based decoding studies have recently provided new insights into the encoding schemes of the human cortex^3^,^4^,^5^,^6^. For the brain, encoding is the process of translating currently perceived stimuli into neural activity patterns. Decoding, its complement, seeks to reproduce already-encoded environmental stimuli via brain activity; it is comparable to downstream processing. Due to the indeterminacy inherent in neural coding in general, successfully mimicking this decoding (defined simply as decoding hereafter), even with the help of full utilization of machine learning techniques, would require knowledge of the complementary encoding scheme. This means, on the other hand, that decoding performance is a barometer of the reliability of the paired encoding scheme^7^, i.e., the neural representation.

We previously reported that scene view anticipation can be decoded from fMRI signals in human fronto-parietal regions^2^, though the neural encoding scheme therein remained uninvestigated. If a population of neural microcircuits were representing a specific scene view, such an encoding system, though relatively inefficient, would be highly robust; even if some microcircuits happened to behave erroneously, the whole population would be able to stably represent the scene view. However, considering the metabolic cost of maintaining a vast number of microcircuits in the brain, there could be a trade-off between efficiency and robustness. Thus, we analysed the data-driven encoding models with a focus on efficient but stable error correction. Additionally, for demonstrating the generalization ability of our encoding models, which are not specific to passive navigation and could generalize to active navigation, we examined cross-task decodability, to see if the decoder constructed by the scene choice task is transferable to the motion decision task.

## Results

### Behavioural results for passive and active navigation

**Figure 1:**
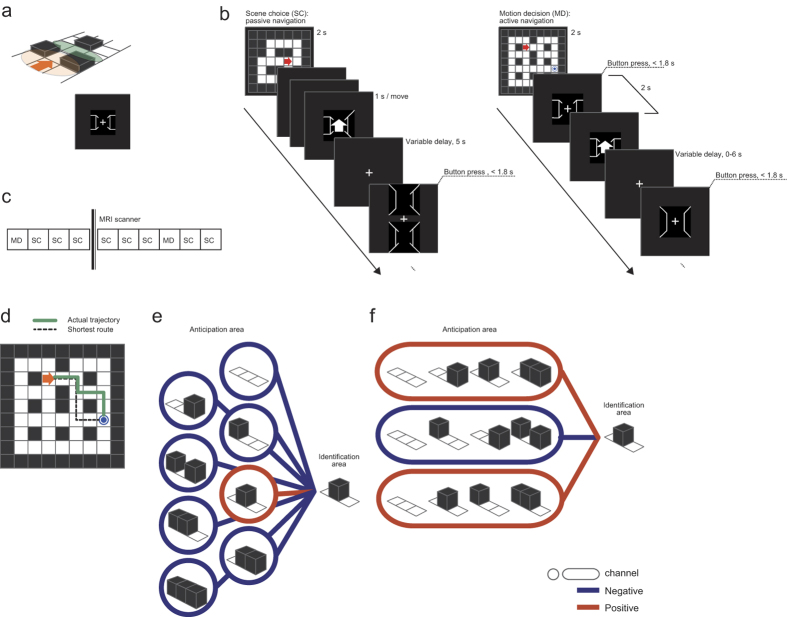
Experimental procedure and encoding scheme. (**a**) A schematic drawing of a single trial in our spatial navigation task. The orange oval indicates the visual field seen by a participant at the state indicated by the red arrow (state = location + orientation); seen are two walls (the forward-left and forward-right view parts) and one path (the forward-centre view part) (bottom). After moving forward, the participant sees three new view parts (green oval). (**b**) Participants took part in scene choice (SC) and motion decision (MD) navigation tasks. In SC (left panel), participants predicted the next scene view consisting of three unseen view parts (forward-left, forward-centre, and forward-right) consisting of either wall (black square on the top display) or path (white square) elements. For each trial, they chose the next scene view from between the correct next scene and an incorrect one. In MD (right panel), participants were requested to navigate from an initial state (red arrow on the top display) to a goal square (blue circle). (**c**) Before the experiment, participants were trained in relating the 2D and 3D views in MD. (**d**) Sample behaviours in MD. Shown are the actual trajectory taken by the participant (green line) and the shortest route (black dashed line). This participant took the second-shortest route to the goal square. Route length was calculated as number of moves including pure rotations. (**e**) We assumed a perceptron architecture, in which multiple, different encoding channels cooperatively represent the next scene view. We had 8 possible scene views, so a naïve encoder design would have eight corresponding channels; when predicting a specific scene view, one channel is activated (red circle), while the others are inactivated (blue circles). (**f**) A more sophisticated encoder. The top and bottom channels (activated) each vote as expecting one of the four possibilities in each red oval, and the middle channel (inactivated) votes as expecting the remaining possibilities in the blue oval. No channel can predict the scene view by itself, but the majority of votes can (here, a predicted wall in the forward-centre view).

Seven participants completed both the scene choice (SC) task and the motion decision (MD) task in an fMRI scanner, during which the participants saw 3D wire-frame views presenting egocentric scenes constructed of open paths and walls (Fig. 1a–c).

In SC, trained participants were requested to choose from two options which scene would appear after a move selected by the computer: the correct upcoming scene or a distractor scene (Fig. 1b, left). We found that participants were able to choose the future scene accurately. Quantitatively, the results were: 93.4% ± 2.6% correct, 5.6% ± 2.3% incorrect, and 1.0% ± 1.0% missed (mean ± SEM across participants; chance accuracy = 50%; see Shikauchi & Ishii^2^ for further details). Incorrect trials were excluded from subsequent analysis, as were missed trials, in which participants did not press the answer button in the allotted time (1.8 s).

In MD, the participants steered to an instructed goal from an initial state over a series of trials, each trial requiring a choice of one of three decision options: move forward, turn left, or turn right (Fig. 1b, right). Experimental blocks were separated by arrival at instructed goal positions. The number of completed blocks was 11.43 ± 0.98 (mean ± SEM across participants), while the uncompleted blocks (final block in each session) were excluded from subsequent analysis. We found that participants could accurately trace the shortest or the second-shortest route (i.e., one or two moves longer than the shortest route, Fig. 1d) in the majority of blocks; the shortest route was traced in 83.7% ± 24.5% of blocks (mean ± SD across participants), and the second-shortest route in 9.6% ± 18.3% of blocks. Reflecting the topology of the MD map, many of the participants’ decisions were forward moves (75.9% ± 9.5%); left and right turns accounted for 12.7% ± 10.0% and 11.5% ± 9.0% of the decisions, respectively. Reaction time (RT) did not differ among these decision types (Friedman test, p = 0.07).

### Comparison between encoding models

**Figure 2:**
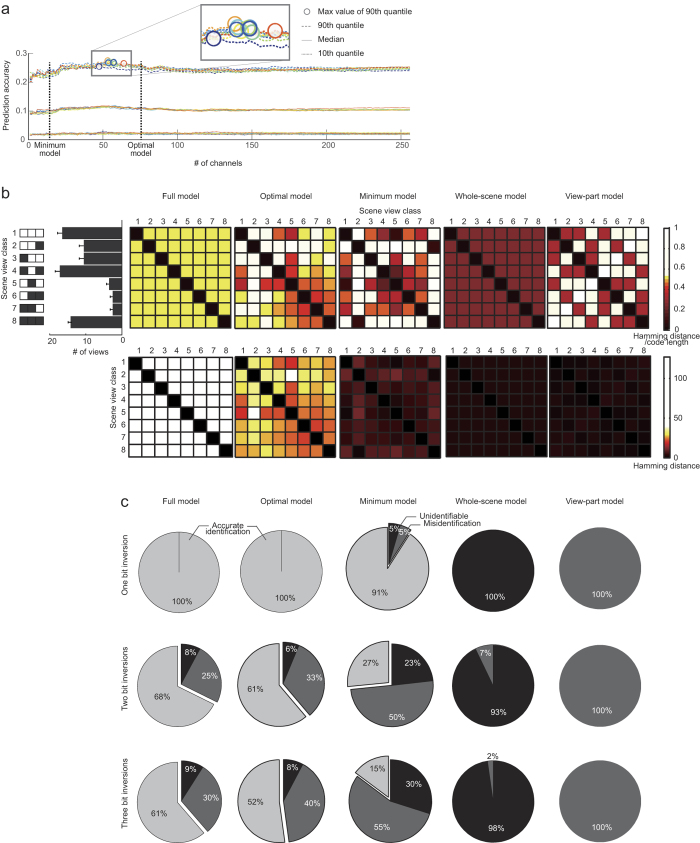
Characteristics of encoding models. (**a**) Voxel-wise prediction accuracy (in terms of Pearson’s correlation coefficient between measured and predicted brain activity) became saturated with 40–70 encoding channels, and did not increase further with larger numbers of channels. A single line corresponds to the prediction accuracy for one validation set from ten folds in the 10-fold cross-validation procedure from a typical individual (participant 5, bilateral IPG). The results of other individuals and ROIs are presented in Supplementary Figure S1. (**b**) Distance matrix in terms of the Hamming distance between pairs of code words of the eight view classes (depicted in the left-most inset; white, path; black, wall), under full encoding (‘Full model’), data-driven optimal encoding (‘Optimal model’), data-driven minimum encoding (‘Minimum model’), naïve eight-class encoding (‘Whole-scene model’), and view-part–wise encoding (‘View-part model’). Top panels indicate the Hamming distance normalized by code length and bottom panels are for the original Hamming distance. The histogram beside the full encoding model shows the normalized frequency of the eight scene views in the SC task. Error bars indicate SD over three different maps. Our data-driven models reflected the scene view frequency, a characteristic of map topography, such that more frequently seen views were more frequently predicted. (**c**) Full encoding and optimal encoding are robust against errors introduced when observing the channels, such that a one bit-inversion error on every encoding channel can be corrected (first row, first and second columns). On the other hand, any single bit-inversion causes unidentifiable codes in whole-scene encoding (fourth column), and leads to misidentification in view-part encoding (fifth column). The second and third rows show the situation when two and three bit-inversion errors occur, respectively. For the other participants’ data, see Supplementary Figure S1.

Next, we sought the encoding model that best encodes the participants’ prediction of the upcoming scene view into their voxel-wise fMRI activity, which was represented by the delay-period activity in the SC task. There were eight prediction possibilities, since each of the three view parts, forward-left, forward-centre, and forward-right, could show only two options (path or wall). Each encoding channel might thus assign ‘1’ (positive) to one or more out of the eight possibilities, and ‘0’ (negative) to the others (Fig. 1e,f); hence, there could be 254 ( = 2^8^–2, all and none were excluded) channels in total, which constitute the *full encoding model* (see *Encoding models*). When these channels were aligned (sorted in descending order) according to their general linear model (GLM) weight value, we found that the multivariate prediction accuracy saturated after incrementally incorporating about 75 aligned channels (Fig. 2a and Supplementary Figure S1, see *General linear model analysis*). This implies that the fMRI signals were expressible by using a smaller number of features, which would be less costly than the full (most expensive) representation.

Our finding that the fMRI signals in SC were well predicted using a small number of encoding channels led to the idea of a *minimum encoding model*. By incrementally incorporating single channels from the aligned channel list sufficient for distinguishing the eight possibilities, we defined the minimum encoding model. As described above, each channel separates the eight scene views into positive and negative groups. As the number of channels increases, the number of identifiable scene views would also increase, as expected. We included channels one at a time, according to the aligned channel list, and stopped adding channels when the number of identifiable views reached eight; this procedure created the minimum encoding model (see Supplementary Figure S2). When using a randomly permuted channel list, the number of channels constituting the minimum encoding model was 6.14 ± 1.68 (the SD was calculated over 5,000 random permutations). However, using our incremental addition paradigm, the smallest number (11.43 ± 2.76; mean ± SD over participants; range 7–15) was larger than when the channels were selected from the randomly permuted channel list.

Any encoding model would assign a binary code word to each of the eight possible views to be predicted; this code is expected to constitute the outputs of the encoding channels. Examining the Hamming distance (number of different bits) between each code word pair of the minimum encoding model in the form of a distance matrix (Fig. 2b, middle), we found that many code words of our minimum encoding model were represented in an idiosyncratic manner. For example, the third class [*wall, path, path*] is distant from its nearest neighbour (the fifth class [*path, wall, path*]), with a Hamming distance of four. Such an isolated class can enjoy error correction in decoding; indeed, even if one or two channel(s) are disturbed by bit-inversion error, the class isolation allows the Hamming decoder to decode this view class accurately. On the other hand, the fifth class [*path, wall, path*] has the first class [*path, path, path*] as its nearest neighbour, with a Hamming distance of one. Although one bit-inversion could thus lead to misclassification in this case, such misclassification occurred only infrequently during navigation, because of the infrequent occurrence of the wall status in the forward view [*, wall, *] in this particular maze environment. By reflecting the characteristics of the current environment, which in this case was the maze topography in SC (Fig. 2b), our minimum encoder realized robustness in its decoding in a data-driven manner. On the contrary, the whole-scene encoding (grandmother cell-like) model, which is the simplest decomposition of a multi-class classification problem into multiple binary classification problems, includes error detection among its characteristics, but cannot correct bit-inversion errors (Fig. 2c, Supplementary Figure S2 and Supplementary Figure S3). It spaces every code word in an equidistant manner, without reflecting any information from the task environment. The most naïve encoding model, the *view-part model*, provides the most efficient representation of the scene views, but has neither error detection nor error correction ability (Fig. 2c); a single bit-inversion error always leads to misclassification.

Although the minimal encoding model included a sufficient number of channels, its error correction ability was insufficient. When we increased the number of channels further, we found not only that the prediction ability of the fMRI signals became stable (Fig. 2a), but also that the decoding ability of the Hamming decoder increased (see *Decoding results*). We called this expanded encoding model the *optimal encoding model* (see *Decoding analysis method*). Our data-driven encoding models reflected the map topography-dependent frequency of the eight scene views (histogram in Fig. 2b, see *Appearance frequency matrix of scene view*) better than data-unrelated, randomly assigned encoding models (optimal encoding model, Pearson’s correlation coefficient = median across participants 0.21, range 0.05–0.56; data-unrelated random encoding model, median −0.01, range −0.61 − 0.61. The Wilcoxon rank sum one-sided test, p < 0.05 × 10^−1^).

### Frontal and parietal cortices are involved in scene anticipation

**Figure 3:**
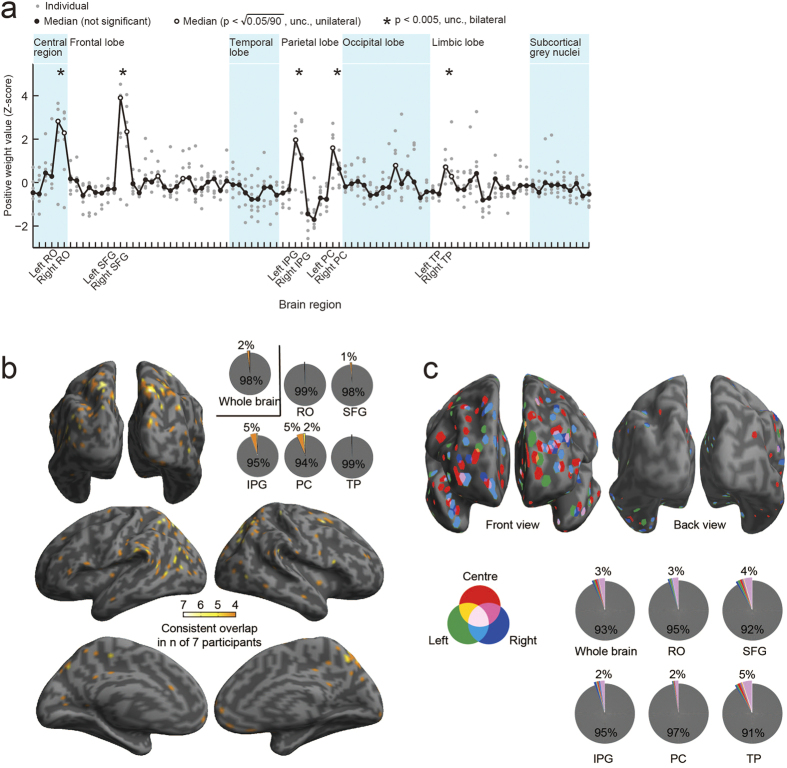
Brain regions involved in scene prediction. RO, rolandic operculum; SFG, superior frontal gyrus; IPG, inferior parietal gyrus; PC, precuneus; TP, temporal pole. (**a**) Mean absolute weight value in the naïve eight-class encoding (whole-scene model, see Fig. 2e). The weight value was normalized across regions of interest (ROIs) within each participant into a Z-score with a mean of 0.0 and standard deviation of 1.0. The abscissa indicates brain ROIs according to a list identified by automated anatomical labelling^31^. Each small, grey dot corresponds to a single participant, and each black, large one to the median of all participants. A unilateral statistical test was applied to 90 brain regions individually. The significance level was set at the square root of 5% with Bonferroni correction for multiple comparisons; if a pair of bilateral regions are independently significant, the pair becomes significant with p < 0.05 (corrected). In addition, a bilateral statistical test was applied to 45 bilateral brain regions with p < 0.005 (unc.). (**b**) Predictable-voxel maps showing overlap of the voxels consistently involved in the whole-scene model, plotted on the inflated brain surface. Bright parts consist of voxels involved in scene prediction in the full encoding model in at least 4 out of 7 participants; a statistical significance threshold of uncorrected p < 0.05 (r > 0.21) was required in each participant. The pie charts show the rates of bright-coloured voxels in the respective brain ROIs. (**c**) Spatial distributions of the voxels contributing to decoding scene predictions. Coloured voxels show those with a statistically significant information gain index (IGI) for at least 3 out of 7 participants, and hence those that would be incorporated into the scene prediction process; the significant voxels (see *Data-driven analysis*) in terms of the IGI are plotted; their colours correspond, respectively, to the different view parts (forward-left: ‘Left’, forward-centre: ‘Centre’ and forward-right: ‘Right’). The pie charts show the rates of coloured voxels in respective brain ROIs.

To identify the cortical regions involved in scene anticipation, we examined voxel-wise weight values in another GLM that reproduced the fMRI blood oxygen level-dependent (BOLD) activities with the whole-scene model. We found substantially large voxel-wise absolute weights consistently across participants in five bilateral brain regions: rolandic operculum, superior prefrontal gyrus (SFG), inferior parietal gyrus (IPG), precuneus (PC) and temporal pole (TP) (Fig. 3a). Although the whole-scene model was used here because the encoding channels in this model were mutually orthogonal, we verified that the above results remained unchanged with use of the full encoding model.

Previous neurophysiological studies have suggested that widespread brain regions, including higher-order fronto-parietal areas, are involved in prediction^8^. When we examined the prediction-relevant voxels, defined as those which exhibited high correlations between measured and predicted brain activity consistently over participants, they were mainly found in the IPG and the PC (Fig. 3b). Figure 3c and Supplementary Figure S4 show the information gain index (IGI, see *Data-driven analysis method*) mapped onto the cortical surface, in which a voxel was coloured when it showed a significant weight value selectively for one of three view parts (forward-left, forward-centre, or forward-right). The SFG and the TP were found to include a large proportion of voxels that showed high information gain irrespective of view part. This result suggested a functional difference in scene prediction between the parietal regions and the SFG and TP; the IPG and PC were involved in scene anticipation itself, while the SFG and TP were specialized for the prediction of specific view parts.

### Decoding results

**Figure 4:**
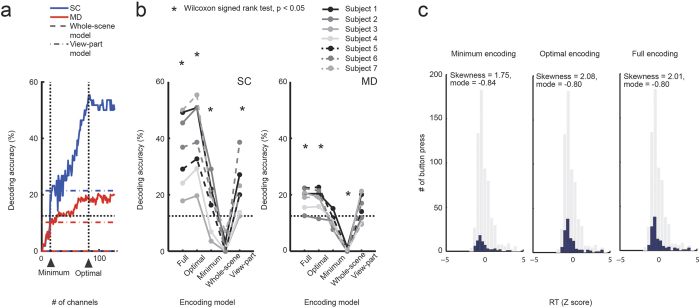
Decoding results. (**a**) Decoding accuracy for an individual (participant 1) against number of encoding channels progressively incorporated into the decoding calculation, starting at the beginning of a list of channels sorted by the general linear model (GLM) weight value. The blue line shows the decoding accuracy validated in the scene choice (SC) sessions (sessions 1, 2, 3, 5, for training, and session 4 for test), and the red line shows the cross-task decoding accuracy for the motion detection (MD) task (the SC sessions for training, and the MD session for test). Dashed lines and dot-and-dash lines indicate decoding accuracies with the whole-scene model and the view-part model, respectively. (**b**) Different encoding schemes lead to different decoding performances in the SC task (left), where each decoding accuracy was examined in the validation session (session 4). Similar tendencies were also observed in the MD task (right). Black asterisks indicate significance (Wilcoxon signed rank test, p < 0.05). (**a,b**) Horizontal, black dotted lines indicate chance level (eight classes, 12.5%). (**c**) When the Hamming decoder was successful in decoding the scene prediction, the participants showed shorter RTs (blue histogram) than those in all decoding trials in MD (grey, skewness = 0.90, mode = −0.63). This RT reduction was most prominent in the optimal encoding model. The RTs were normalised to have mean zero and variance one within each participant.

For each channel constituting any encoding model, we trained a linear support vector machine (SVM) to output positive (‘1’) or negative (‘0’) based on the delay-period brain activities in SC. As the number of encoding channels increased, the accuracy of the Hamming decoder (see *Decoding analysis method*) was likely increased (Fig. 4a and Supplementary Figure S5) in accordance with the Condorcet jury theorem^9^. However, the full encoding model employing all 127 channels (=254/2, considering the symmetric nature of SVMs) did not show the highest decoding accuracy. The minimum encoding model did not show sufficient decoding accuracy either, due possibly to a lack of the necessary features (encoding channels). As a moderate choice, we defined an optimal encoding model that showed the highest decoding accuracy in SC, which was in turn evaluated using the MD task (cross-task validation). The decoding accuracy of this optimal encoding model was comparable to or even better than that of the full encoding model in MD (paired Wilcoxon signed ranks test, p = 0.88) (Fig. 4b). We also found that the decoding performances of the whole-scene model (eight channels) and the view-part model (three channels) were almost zero and at chance level, respectively. The decoding accuracy in MD of the optimal encoding model was significantly higher than chance and that of the view-part model (Wilcoxon signed ranks test, p < 0.05). Accordingly, the whole-scene model and the view-part model showed no cross-task decodability for scene view prediction, while our optimal encoding model allowed us to decode the upcoming scene views based on fMRI brain activities. The model was able to fully utilise the brain signatures of scene prediction during navigation, which we found to be represented by multiple channels in a distributed manner.

Next, we divided all the MD scans into those in which participants pressed a button (decision scans) and all others (delay scans). Examining the relationship between decoding outcomes based on fMRI activities during decision scans and the RTs in the trials covering the decision scans, we found that participants showed shorter RTs when the decoding outcome for the scene prediction was more successful than those of all MD trials (Fig. 4c). Moreover, this reduction in RT was most prominent when employing the optimal encoding model. On the other hand, there was no significant difference in the decoding accuracy between the decision scans and the delay scans, regardless of the employed encoding models (Wilcoxon matched-pair signed ranks test: minimum model, p = 0.81; optimal model, p = 0.58; full model, p = 0.94). Accordingly, there was a tendency for the decoding to be successful when the decision RT was relatively short.

**Figure 5:**
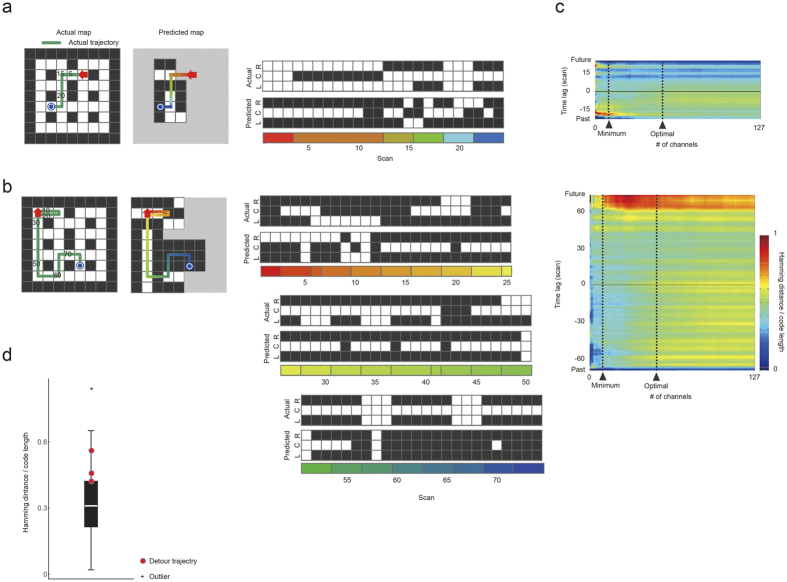
Sequential decoding in two motion decision task (MD) blocks. (**a,b**) Scene reconstruction from single-scan fMRI activity (2 s) over a single MD block, but before this participant (participant 1) saw the actual scene. The left panel indicates the trajectory that was taken by the participant, starting from an initial state (red arrow) to a goal (blue circle). A digit on the map shows the scan index corresponding to the horizontal axis in the middle panel. The middle panels indicate the series of correct scene views (upper) and predicted scene views (lower). The hemodynamic response delay was not compensated for in this decoding analysis. L, forward-left; C, forward-centre; R, forward-right. (**c**) Time shift Hamming distances, which signify the discrepancy between a real scene (not necessarily the present one) and the predicted one decoded from current brain activity. The top and bottom panels correspond to the MD blocks in **(a)** and (**b**), respectively. (**d**) Distance between the code words predicted for the scenes around the goal position based on the initial three scans and the code words of the corresponding true scenes. The optimal encoding model was used. In the blocks with detour trajectories, the distance was longer than the median of the other trajectories (white line). The bottom and top edges of the box indicate the 25^th^ and 75^th^ percentiles, respectively. A point located beyond 1.5 times the box height was defined as an outlier.

Although the participants had been requested to choose the correct next scene, making prediction of the next scene crucial to the SC task, this was not necessarily the case in the MD task, because the MD participants were requested to reach their destinations with as small a number of trials as possible. Actually, the participants showed good planning performance in MD (see *Behavioural results on passive and active navigation*). Thus, anticipation was not limited to one-step prediction. To investigate the decodability of two or more steps of anticipation, we examined a time-shifted Hamming distance (TSHD), which measures the distance between the predicted code word based on current brain activity and the correct code word for a future or past scene view with some lag time. Interestingly, the TSHD did not show a minimum value with a lag time of 0 (one-step prediction); that is, smaller was not always better (see Fig. 5a,b and Supplementary Figure S6). In particular, when the participant’s behaviour was very successful, the TSHD showed minimum values for future scene views that were close to the goal (Fig. 5a and b). On the other hand, the blocks with a detour trajectory (defined as a trial where the number of steps exceeded the shortest route by 10 or more steps) had a larger TSHD between decoded code words in early steps, and those of real scene views around the goal (Fig. 5c).

## Discussion

In our encoding/decoding analyses of fMRI activities during the performance of two types of 3D navigation games, we made two major observations. (i) Although our data-driven minimum and optimal encoding models were defined so as to exclusively and accurately reproduce the participants’ brain activities, they also identified encoding channels that were effective in robustly transmitting scene anticipation. These encoding models allowed the simple Hamming decoder to work well even when the channels suffered from noise, because they reflected the characteristics of the navigation environment. They also allowed us to examine specific brain regions that can be involved in those encoding models. (ii) By using such data-driven encoding models, we realized not only better decoding ability than the naïve encoding models, but also reasonably good cross-task decodability. Actually, our encoder/decoder pair was obtained only from data recorded during passive navigation (i.e., the SC task), but showed significant decoding ability even when applied to the data recorded during active navigation (i.e., the MD task).

The encoding strategy of the brain, especially for its high-dimensional cognitive states, is comparable to the problem of feature representation in the field of machine learning. Error-correcting output coding (ECOC) provides a general framework for representing high-dimensional features in a robust manner^10^,^11^,^12^. According to ECOC, an original multi-class classification problem is decomposed into multiple binary classification problems; each of these then acts as a transmission channel, transmitting the message of the original classes even if it may be noisy. When using a larger number of transmission channels than the number of original multiple classes, a receiver can detect an original message based on received codes that have passed through noisy transmission channels. Thus, the ECOC provides a powerful encoding scheme to allow robust information transmission through multiple but unreliable channels. In this study, we presented data-driven encoding models based on ECOC. There are three major reasons for the plausibility of ECOC-based encoding models in the brain. First, neural information pathways (e.g., axons) are probabilistic rather than deterministic, due to the stochastic nature of neuronal spikes and the instability of axonal transmssion^13^. Thus, the neural decoding system inevitably needs robustness to oppose the probabilistic factors (noise) involved in its transmission system. Second, since each neural element (neuron) can carry binary information (spike or non-spike), the network state at a single moment is that of a binary vector. One natural scenario to solve a multi-class problem by using such a binary system is to decompose the multi-class problem into multiple binary problems, i.e., ECOC. Third, the brain has massively parallel processing machinery^14^,^15^, leading to the idea that its encoder could consist of massively parallel but noisy, channels. On the other hand, in ECOC, there is a trade-off between communication cost and ability to correct errors; an increase in the number of channels is good for robustness against noise, but comes at the high cost required to both prepare the physically necessary channels, but also to biochemically activate those channels for transmission. Our minimum and optimal encoding models significantly reduce this trade-off problem, by selecting channels essential for the current environment in a data-driven manner, as described below.

During navigation, individuals perform path planning, in which the question of where to move is more important than whether or not they can move. Thus, learning the locations of paths is more crucial than the locations of walls, to allow the participants to identify available directions. The minimum and optimal encoding models, representing participants’ fMRI activities, had a tendency to assign active representation (i.e., ‘1’) to scene views including paths (see Supplementary Figure S2). Moreover, the minimum and optimal encoding models allocated their code words so as to make the four scene views whose centre square was a path more robust against bit inversion than the four views whose centre square was a wall (Fig. 2b and Supplementary Figure S2). These results suggest that our minimum and optimal encoding models acquired a feature representation suitable for a subsequent decoding process by means of noisy information transmission; a similar encoding scheme could be implemented in the brain to produce stable decision making by decoding noisy information provided by its upstream channels. We also found that our data-driven minimum encoding model needed more channels than did a data-unrelated random encoding model that incorporated channels in a random order. This probably occurred because the GLM assigned larger beta weights to more important features. Such features, represented as encoding channels, would be placed with a high ranking according to our method. The more important features were likely correlated with each other, increasing redundancy in the list relative to that of the randomly ordered list. On the other hand, such redundancy in our data-driven encoders led to higher decoding performance versus that of the data-unrelated random encoders. This analysis suggests that our data-driven encoding models could detect important features, by paying for redundancy, and were effective for robust decoding against bit-inversion errors that can occur when decoding from noisy brain activities (Fig. 2c and Supplementary Figure S3).

The frontal and/or parietal regions have been thought to be possible sources of anticipation in two contexts, reward prediction and future path planning^16^, ^17^, ^18^, ^19^, ^20^, ^21^. In the present study, we found that the GLM weights with the whole-scene model exhibited large values in five bilateral brain regions (Fig. 3a): rolandic operculum, SFG, IPG, precuneus, and TP. Moreover, the voxels showing a higher information gain index were localised to the SFG and TP, and those showing higher prediction accuracy were localised to the IPG and precuneus (see Fig. 3b–c and Supplementary Figure S4). These results suggest that the fronto-parietal regions are involved in general aspects of scene anticipation, not just in reward prediction or path planning. Although the rolandic operculum was also identified in the GLM weight-based analysis, we found no further evidence that this region is related to encoding scene anticipation.

Although we did not directly investigate brain activities involved in path planning, scene anticipation should accompany path planning^22^,^23^, especially in active navigation tasks such as MD. Actually, the decoding performance slightly increased over the progress of active navigation in MD (Fig. 5a), and scene anticipation was more accurate when a participant’s performance was better. Moreover, when the participants followed a longer route than the optimal/suboptimal one, the predicted code words for scene views in the present route were similar to the correct code words of the scene views that they had experienced in the past (Fig. 5b and Supplementary Figure S6). These results imply that participants sometimes got lost, and could no longer predict upcoming scene views as far into the future. Furthermore, the observation that the future scene views could be predicted from current brain activities (Fig. 5a, upper), especially when the participants were performing well in MD, may indicate that the participants’ brain activities comprised prediction factors over multiple future steps. Such factors related to path planning might have lowered the decodability of one-step future scene views during active navigation in MD in comparison with passive navigation in SC (Fig. 4b).

Existing fMRI decoding studies presented encoding models embodied in higher visual areas^4^,^5^,^6^,^24^,^25^. Our present study focused on scene anticipation, which could be related to visual processing but is hypothetically a downstream visual process, and revealed that encoding can be localised in frontal and parietal areas that are known to be involved in decision making. We know very little of how information is represented for decision making, especially in higher brain regions. This lack of knowledge has motivated us to develop our data-driven ECOC encoders, the minimum and optimal encoding models, as particular implementations of a general error-tolerant encoding scheme. Our analysis of robustness suggested that our data-driven encoding models could have detected important features, thanks to their higher redundancy relative to the random channel list-based encoding models or the model with absolutely minimum number of channels (view-part model).

Previous studies have suggested that the human brain adopts parallel processing in the perceptual and behavioural stages, but serial processing in the decision stage^12^,^13^. The phenomenon of anticipation occurs midway between the perceptual stage and the step of behaviour selection, and hence is considered to be in the early decision stage. The finding here that our optimal encoding model was more plausible than its deterministic counterparts, the whole-scene and view-part models, might suggest that scene anticipation is also processed in parallel so as to be error-tolerant. Notice, however, that fMRI has limited temporal resolution due to hemodynamic delay, so there remains the possibility that anticipation actually depends on serial processing. Meanwhile, the Bayesian brain hypothesis has been widely discussed in a number of studies^26^,^27^,^28^. This hypothesis assumes that the brain manipulates multiple possibilities by maintaining a probabilistic distribution (prior) of them. Our current study is theoretically compatible with these studies.

We constructed the data-driven encoding models by aligning the channels based on the beta weight values. Moreover, we used multiple regression with the rank-deficient design matrix ***X***^***full***^ (see *Encoding models*). When the design matrix is not full rank, the meaning of beta values becomes obscure. Considering this issue, we used the full rank design matrix ***X***^**1*****R***^ when making the cortical map of scene view anticipation (Fig. 3a). On the other hand, we relied on the multiple regression with the rank-deficient design matrix (i.e., the full encoding model) when aligning the channels to construct data-driven encoding models. The beta weights in the multiple regression provided us with a systematic way to estimate the contributions of channels onto brain activities, even though they are mutually correlated. Even with redundancy coming from such mutual correlation, the decoder complementary to the data-driven optimal encoder showed high decoding accuracy. Although we cannot say that our brain really utilizes such redundancy in its encoding scheme, we can say at least that the redundancy was indeed effective to extract information from, possibly redundant, brain activities.

Our encoding models combined multiple binary channels linearly for representing fMRI activities. These constraints, linear combination and binarization, allowed us to describe our encoding models by ECOC. However, there may be more sophisticated encoding models in non-linear and/or non-binary domains. Seeking and describing such advanced encoding models would facilitate our understanding of the encoding mechanism of scene anticipation. Furthermore, scene anticipation in unfamiliar situations remains to be understood. The hippocampal place system contributes to the encoding of not only familiar situations, but also to a related novel experience occurring in the future^29^. Are such ‘preplay-like’ activities involved in scene anticipation mediated by the cerebral cortex? If yes, our decoding method may be able to detect individual expectations depending on his/her *a priori* knowledge or character. Additionally, although all our analyses in this study were performed offline, the decoding analysis in the MD task can be extended to the online mode with the help of online fMRI measurement techniques; our encoder and decoder were solely determined by data from the SC task, which could be completed in advance of the MD task. Such online decoding technology may lead to the development of new brain-based devices to assist human navigation and decision-making, for example, a secure guidance system that warns users of upcoming dangerous behaviours by notifying them of an incongruous belief that does not match reality.

## Methods

### Participants

Eight healthy participants (1 female author, 7 males; aged 21–28 years) took part in this experiment. Each participant gave written informed consent, and all experiments were approved by the Ethics Committee of the Advanced Telecommunications Research Institute International, Japan. All methods were carried out in accordance with the approved guidelines.

### Data acquisition

A 3.0-Tesla Siemens MAGNETOM Trio A Tim scanner was used to acquire interleaved T2*-weighted echo-planar images (EPI) (TR = 2 s, TE = 30 ms, flip angle = 80°, matrix 64 × 64, field of view 192 × 192, voxel 3 × 3 × 4 mm, number of slices 30). A high-resolution T1 image of the whole head was also acquired (TR = 2,250 ms, TE = 3.06 ms, flip angle = 90°, field of view 256 × 256, voxel 1 × 1 × 1 mm).

### Experimental procedure

The participants performed two types of 3D spatial navigation tasks on the same day, one of which, the scene choice (SC) task, has been described previously^2^ and the other, the motion decision (MD) task, is new to this study.

#### Pretraining

On the day before scanning, the participants performed practice tasks to memorize the map topography and to become familiar with the relationship between the 2D maps and the 3D views of the maps, according to the procedure reported previously^2^. In the first practice task, the participants performed a free navigation task in a map with 9 × 9 squares of white paths and black walls, which was the same map as would be used in the MD task on the subsequent day. After observing the current state on the 2D map and the 3D wire-frame view seen at the current state simultaneously, they indicated their chosen movement using one of three keys on a computer keyboard to choose ‘go forward’, ‘turn left’, or ‘turn right’. This practice task continued until the participants reported that they were familiar with the 2D-3D association. Then participants performed a practice SC task with three 7 × 7 maps, which were the same maps as would be used in the SC task on the subsequent day. This second type of practice continued until the participants could choose the correct upcoming scene with more than 80% accuracy.

#### Scene choice task

The following day, participants performed the experimental tasks in the scanner. The SC task consisted of 320 trials, spread out over five sessions (64 trials per session). The sessions were separated by breaks of a few min each. Between the third and fourth sessions, another experimental task, the MD task, was performed. An SC trial consisted of four periods: a map period (2 s), a move period (three moves, 1 s/move), a delay period (5 s) and a choice period (1.8 s). In the map period, participants were presented with an initial position and orientation on one of three 2D maps (Fig. 1b). During the move period, the participant’s state was moved three steps by the computer. Hence, they passively viewed a sequence of 3D scene views, each accompanied by a pre-indication of the next move direction. Visual feedback indicating the next direction was presented as a white arrow on the centre of the screen. ‘Up arrow’ indicated forward movement, and ‘left arrow’ or ‘right arrow’ indicated left or right turns, respectively, while staying at the same square.

The first and second movements could be any of the three movement types (move forward or turn left/right), while the third movement was constrained to be forward. Before the third movement but after a pre-indication of the third move was presented, a delay period was introduced.

In the following choice period, participants were requested to choose as soon as possible, using an MRI-compatible button box, between two options, a correct scene, and a distractor scene, both represented in 3D wire-frame format. By their choice, participants were to indicate which scene they expected to see after the third movement. The new 3D scene after the third and forward movement contained three newly observed view parts (forward-left, forward-centre, and forward-right), which had never been seen in that trial in the preceding move period or in the delay period.

#### Motion decision (MD) task

In the MD task, the participants navigated the same 9 × 9 map as was used in the previous practice task (Fig. 1d). A single block started with presentation on the map of the initial state and the goal position and ended with goal achievement. At the onset of a block, the initial state (position and body orientation) and the goal position were presented on a screen for 4 s. At each trial, a 3D wire-frame scene view at the current state was displayed and the participant was requested to make a decision as to how best to reach the goal, by pressing one of three action buttons, ‘go forward’, ‘turn left’, or ‘turn right’, of a three-key MRI-compatible button box, within a fixed decision period of 1.8 s, but as soon as possible. After the participant made a decision, visual feedback indicating the selected direction was presented as a white arrow on the centre of the screen (feedback period, >0.2 s). After a fixed time consisting of the decision period and the feedback period (2 s in total), a variable delay time of 0–6 s was imposed, then a new trial was started by displaying the next scene view.

After goal achievement, the next block was immediately started. While the combinations of initial states and goal positions were different between blocks and their orders were further different between participants, they were extracted from a predefined pool that was common over the participants. The MD session comprised a fixed number of 300 MR scans and admitted termination even in the middle of a block; only the data for completed blocks were used for analyses.

Since one of the eight participants did not complete both the SC and MD tasks, further analyses were performed only on the remaining seven participants.

### Behavioural analysis

All behavioural data analyses were performed with Matlab (MathWorks) with the help of the Statistics and Machine Learning toolbox. When searching for the shortest route in each MD block, we used Dijkstra’s algorithm implemented as a custom program in MATLAB. Reaction-time analysis was performed after conversion of the RT data to within-subject Z-scores.

### FMRI data preprocessing

The first six scans of each run were discarded so as not to be affected by initial field inhomogeneity. The acquired fMRI data underwent 3D motion correction using SPM12 (http://www.fil.ion.ucl.ac.uk/spm). The data were coregistered to the whole-head high-resolution anatomical image, and then spatially normalized to the MNI space (Montreal Neurological Institute, Canada). We then identified the 37,262 voxels of the cerebral cortex. The fMRI signals then underwent quadratic polynomial trend removal and noise reduction by means of singular-value decomposition (K = 3), and were temporally normalized within each session.

### Encoding models

In our experimental setting, each scene view to be predicted could be represented by the path/wall status of three view parts, forward-left, forward-centre, and forward-right, and thus represents one of eight possibilities (2^3^ = 8). Each possibility is called a class. In our encoding scheme, each class out of the eight possibilities is represented as a code word, a string of binary codes (‘0’ or ‘1’) of which each corresponds to a single channel (Fig. 1e). More specifically, a single channel is represented as a binary column vector **z **= [*z*_1_ … *z*_8_]^***T***^. Each element is either 0 or 1, and the subscript indicates a single class out of the eight possibilities. By concatenating *M* channels, we obtained a code matrix of an encoding model **Z**, which is an 8 × *M* matrix. When the *m*-th element of a code word corresponding to class *c* is 0, *z*_*c,m*_ = 0, the fMRI activities through this channel are assumed not to change regardless of the scene view being class *c* or not. When *z*_*c,m*_ = 1, on the other hand, the activities are assumed to be different between scene views of class *c* and the other views. The design matrix, also called an encoding model, for regressing the fMR activities during the navigation task, was given as an *N* × *M* matrix, where *N* is the number of fMRI scans; each row vector was a single code word that represents the scene view class to be predicted. Below, we will define three encoding models: the whole-scene encoding model (*M* = 8), the view-part encoding model (*M* = 3) and the full encoding model (*M* = 254).

#### Whole-scene encoding model

The simplest way to distinguish eight classes is to use an eight-bit representation, each bit designating one scene view (sometimes called one-versus-the-rest encoding; 1R); the code matrix *Z*^*1R*^ becomes the 8 × 8 identity matrix. The 1R encoding model *X*^*1R*^ ([*N* × 8], *N* = number of samples) is defined as 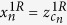; the left-hand-side is the *n*-th row vector of *X*^*1R*^ and the right-hand-side is the *c*_*n*_-th row vector (code word) of the code matrix *Z*^*1R*^, where *c*_*n*_ is the index of the known scene view to be predicted.

#### View-part encoding model

The view-part encoding model is the same as the original three-bit representation of the scene view. Then, the code matrix of the bit-wise encoding *Z*^bit^ is an 8 × 3 matrix. Every column has four ‘1’s and four ‘0’s (Fig. 1f). The view-part encoding model *X*^bit^ is an *N* × 3 matrix, whose row vector is the bit-encoding code word of the scene view to be predicted for the corresponding data point.

#### Full encoding model

Since each encoding channel assigns ‘1’ to one or more scene views out of eight candidates and ‘0’ to the others, there are in total 254 (2^8^–2) possible meaningful channels, excluding all ‘0’s or all ‘1’s (i.e., all or none). For each scene view, a 254-dimensional vector consisting of the binary assignment over these 254 channels becomes its code word in the full encoding model. The code matrix of full encoding, *Z*^full^, is then an 8 × 254 matrix. The full encoding model *X*^full^ is then represented as an *N* × 254 matrix, whose row vector is the full-encoding code word of the scene view to be predicted for the corresponding data point. Full encoding includes all the encoding channels in the whole-scene model.

### General linear model analysis

To perform data-driven analyses, which will be explained below, we estimated general linear models (GLMs) for the full encoding model *X*^*full*^ [*N* × 254], using as input the delay-period fMRI activities in sessions 1, 3, and 5 of the SC task. The GLM optimized the beta weights for the 254 channels to make them fit to the fMRI data over all the training trials, according to the multiple regression analysis. A code word, a 1 × 254row vector of *X*^full^, defined a binary representation of a particular class of a scene view, out of its eight possibilities. Although the number of explanatory beta values is fairly large, 254 × *V (V* = the number of fMRI voxels), they could be determined, using further number of fMRI activities, by the L2-regularized least squares method. In this method, the regularization hyperparameter was tuned by applying a 10-fold cross-validation procedure to the training dataset, searching from 2 to 2^20^ with a logarithmically scaled interval. Six nuisance parameters that remove motion artefacts due to realignment were added to the GLMs. To avoid confounding any activation related to the visual presentation with button-press–related activity, no hemodynamic response function was convolved onto the fMRI signals.

When examining brain regions involved in prediction of upcoming scene views, we used another GLM whose design matrix was full-rank and corresponded to the whole-scene model (Fig. 3a; see below).

### Data-driven analysis

Data-driven analyses on the different encoding models were performed by using the estimated GLM weight values. To examine the plausibility of different encoding models, we introduced two measures: the predictability of the fMRI BOLD signals based on the GLM weights, and the amount of error-correction ability attributable to the code matrix. GLM weights were further used to investigate the cortical correlates of scene prediction.

#### Predictability of encoding models

We examined the voxel-wise Pearson’s correlation coefficient between measured fMRI signals and fMRI signals reconstructed by the GLM whose design matrix was defined as corresponding to the full encoding model. When displaying the prediction accuracy (Fig. 2a), we used the 10th and 90th percentiles and median, rather than the mean, of voxel-wise correlation coefficients over the cortical voxels in each ROI, to reflect the intracortical localization of cerebral functions.

Figure 2a shows the prediction accuracy for each fold in the 10-fold cross-validation when the GLM used a code matrix with different numbers of channels taken from the channel list of the full encoding model.

#### Appearance frequency matrix of scene view

We examined how robustly the set of code words in each encoding model were represented by reflecting the maze topography. When navigating a maze, information regarding scene anticipation should be transmitted more robustly for views that are more frequently seen in the maze versus those less frequently seen. We first constructed an appearance frequency matrix of scene view, i.e., an 8 × 8 matrix ***a***, whose element *a*_***c,c′***_ was the minimum of appearance frequency values of scene views *c* and *c*′. Here, the appearance frequency value was a normalized element value of the view histogram in Fig. 2b, left. This appearance frequency matrix does not depend on any encoding model. To measure the robustness of an encoding model, we calculated the Pearson’s correlation between the off-diagonal part of the encoder’s distance matrix (Fig. 2b, upper right) and the corresponding part of the appearance frequency matrix. A higher correlation implies the encoder’s higher robustness against bit-inversion errors which reflects the map topography; if a particular scene is frequently seen in the maze, the corresponding code word should be isolated (then, error-tolerant), associated with larger element values in the distance matrix, while another scene that is less frequently seen can be associated with smaller element values in the distance matrix (less error-tolerant, but errors barely occur).

When examining the similarity between the appearance frequency matrix and the distance matrices of random encoding models, we prepared 1000 random encoding models, each with 83 channels randomly taken from the full encoding channel list; the number of channels therein was fixed at the median number of the optimal encoding models over participants.

#### Robustness of encoding models

Consider an encoding process in which each encoding channel tries to transmit either of two symbols, ‘0’ or ‘1’. Due to the presence of noise, or more precisely, bit-inversion error, a transmitted ‘0’ may sometimes be received as ‘1’, or vice versa. If there is no prior information available about these transmission channels, the most reasonable way to decode from multiple noisy channels is Hamming decoding, which selects the class whose correct code word is of minimum Hamming distance (Hamming distance = the number of bits different between the two binary code words) from the ‘predicted’ code word. In this study, the latter was defined as the vector aligning the outputs from binary classifiers, each corresponding to a single encoding channel. The results of this analysis are presented in Fig. 2b and Supplementary Figure S1.

Another criterion for the robustness of each encoding model is the capability of error correction. To quantify this, we applied every possible bit-inversion error to each of the eight code words and evaluated how well the Hamming decoding worked. When the Hamming decoding could lead to the right class, because the code word suffering from the bit inversion still fell at or below the minimum Hamming distance from the correct code word of the right class, the trial outcome was classified as ‘accurate identification’. If decoding led to a wrong class, the outcome was classified as ‘misidentification’. The remaining possibility, where the bit inversion made the noisy code word equidistant from correct code words of two or more classes, was called ‘unidentifiable’. We can define these three categories even when each code word is disturbed by simultaneous inversions of two or three bits. If the proportion of the first category ‘accurate identification’ is large, the corresponding encoding model is said to have the characteristic of error correction. The results of this analysis are presented in Fig. 2c and Supplementary Figure S1.

#### Characterizing information gains

An information gain^30^ is the change in the information entropy, an average achieved by getting information through a single encoding channel. The information gain *gain(q)* of channel *q* was defined as


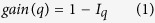



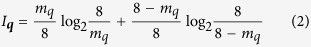


*I*_*q*_ quantifies the information (in bits) that would be obtained by observing a single channel *q*, if the observation is noiseless, and *m*_*q*_is the number of code ‘1’s in this channel *q*. If there is no *a priori* information regarding a single view part, the information entropy is 1 ( = log_2_ 2), because there are two equal possibilities (path/wall). The information gain represents how much this initial entropy is reduced, so it falls within [0, 1]: 1 indicates that the channel provides certain information (without ambiguity) of the status of the view part; 0 indicates that the channel provides no information.

For the three view parts, forward-left (L), forward-centre (C), and forward-right (R), we calculated the information gain *gain(q),* represented as *gain(q)*_*L*_, *gain(q)*_*C*_, and *gain(q)*_*R*_, respectively. To comprehensively represent the information gain over all possible channels, we computed an information gain index (IGI) that quantifies the overall contribution of a specific cortical voxel to the prediction of each view part:


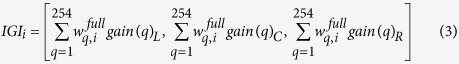


where *w*^*full*^ is the absolute weight of the GLM constructed from the full encoding model and *i* is the voxel index.

When mapping onto the cortical surface, we applied voxel-wise *t*-tests. The null hypothesis was that the IGI comes from a normal distribution with a mean of 0.13 (mean of the above-defined *gain* over channels) times a baseline activity (mean activity of all scans), and with unknown variance. Figure 3c shows the significant voxels in terms of the IGI (p < 0.1).

### Decoding analysis method

#### Training of scene anticipation classifiers

For each channel of an encoding model, a binary classifier called a linear support vector machine (SVM) was trained, using the SC sessions 1, 2, 3, and 5 over the whole cortex. Based on the fMRI voxel-wise signals and the set of code words defined by the encoding model, a set of SVM classifiers (whose number is the same as the number of encoding channels) was trained, to be ready for decoding. Given input voxel values **y** = [y_1_, … y_v_] (*V* is the number of voxels), SVM provided a discriminant value for classifying between scene view classes assigned a label ‘1’ and those assigned a label ‘0’, based on the code matrix of the encoding model. Considering the relatively small number of samples available for training and testing, we did not tune the ‘C’ constant in the SVM (C = Inf.), as previously described^31^.

It should be noted that each SVM classifier was invariant in its function over exchange of the labels ‘0’ and ‘1’, and that the Hamming decoding was invariant over permutation of channel order. Thus, in the decoding analysis, we redefined the full encoding model to remove unnecessary channels; when selecting one channel from the set of equivalent ones, we selected the earlier one on the channel list aligned with respect to GLM weights (see *Comparison of encoding models*). Additionally, we introduced a subsampling scheme for removing imbalances in the number of training samples assigned ‘1’ (positive) and ‘0’ (negative); simple use of an unbalanced dataset to train binary classifiers may introduce a bias into the classifiers. Let *N*_pos_ and *N*_neg_ be the numbers of positive and negative samples in the original training dataset, respectively. If *N*_neg_ > *N*_pos_, then *N*_pos_ negative samples were randomly subsampled, otherwise *N*_neg_ positive samples were subsampled, so that the numbers of positive and negative samples in the training dataset became equal.

When decoding the next scene view from the set of SVM binary classifiers, we used a Hamming decoder. According to Hamming decoding, the output is the one class out of the eight possibilities whose code word (which depends on the encoding method) has the smallest Hamming distance from the predicted code word, the latter being the set of binary outputs from the SVM classifiers. It should be noted we did not use any prior information such as map topography to supplement the participant’s brain activities.

#### Scene reconstruction

When visualizing the predicted scene view during active navigation, we first calculated the wall probability vector in the scan *i, P*(*v*_*i*_), based on a vote of the outputs from the SVM classifiers.


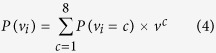



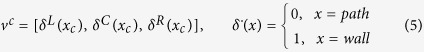



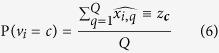


where *Q* is the number of encoding channels (same as the code length). Then each element *P*(*v*_*i*_) had an analogue value, ranging between 0 and 1. To regulate the inhomogeneity of basal brain activities, we binarized each element of *P*(*v*_*i*_) to obtain the predicted scene view (path/wall) by applying a block-dependent threshold set to the within-block median of *P*(*v*_*i*_). Figure 5a and b show the results.

#### Time-shift Hamming distance

To investigate the decodability of two or more steps of anticipation, we defined the time-shift Hamming distance *D* as the Hamming distance between the predicted code word 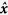, which is the alignment of the SVM’s outputs based on current brain activity, and the code word ***x*** for a correct, possibly future, scene view with a lag time *j*:


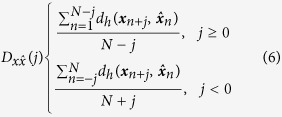


where *d*_*h*_(***x**, **y***) is the Hamming distance between ***x*** and ***y***. Although we were mostly interested in decoding future scene views, we also examined the decodability of past scene views; in the past case, the lag time *j* was a negative integer. The results are shown in Fig. 5c and Supplementary Figure S6.

## Additional Information

**How to cite this article**: Shikauchi, Y. and Ishii, S. Robust encoding of scene anticipation during human spatial navigation. *Sci. Rep.* **6**, 37599; doi: 10.1038/srep37599 (2016).

**Publisher’s note:** Springer Nature remains neutral with regard to jurisdictional claims in published maps and institutional affiliations.

## Supplementary Material

Supplementary Figures
