# Supplementary material for: Robust encoding of scene anticipation during human spatial navigation
Source: Sci Rep. 2016 Nov 22;6:37599. doi: 10.1038/srep37599 (PMC5118749; doi:10.1038/srep37599)
Supplement: Supplementary Figures [file srep37599-s1.doc]

Supplementary Figures

for

‘Robust encoding of scene anticipation during human spatial navigation’

Yumi Shikauchi^1,2^, Shin Ishii^1,2^

^1^Graduate School of Informatics, Kyoto University, Kyoto 606-8501, Japan

^2^ATR Cognitive Mechanisms Laboratories, Kyoto 619-0288, Japan


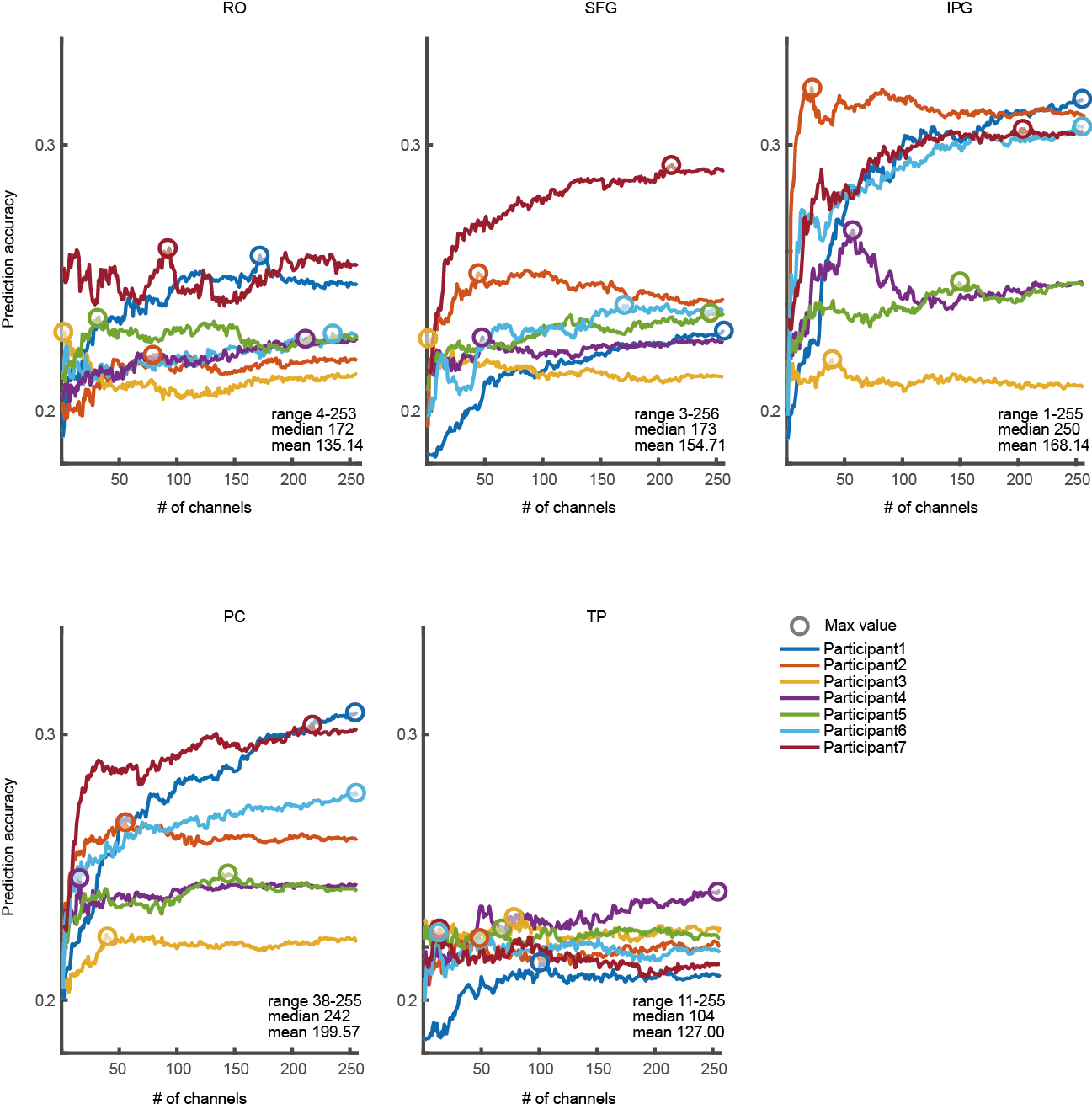
Supplementary Figure S1. Averaged prediction accuracy over ten folds in the 10-fold cross-validation in each individual. Each line indicates the mean prediction accuracy of the top 10^th^ percentile in each region of interest (ROI). The range, median and mean (over individuals) channel numbers needed for maximum prediction accuracy when using the top 10% of ranked voxels in each ROI are shown as the right-bottom legend.

**
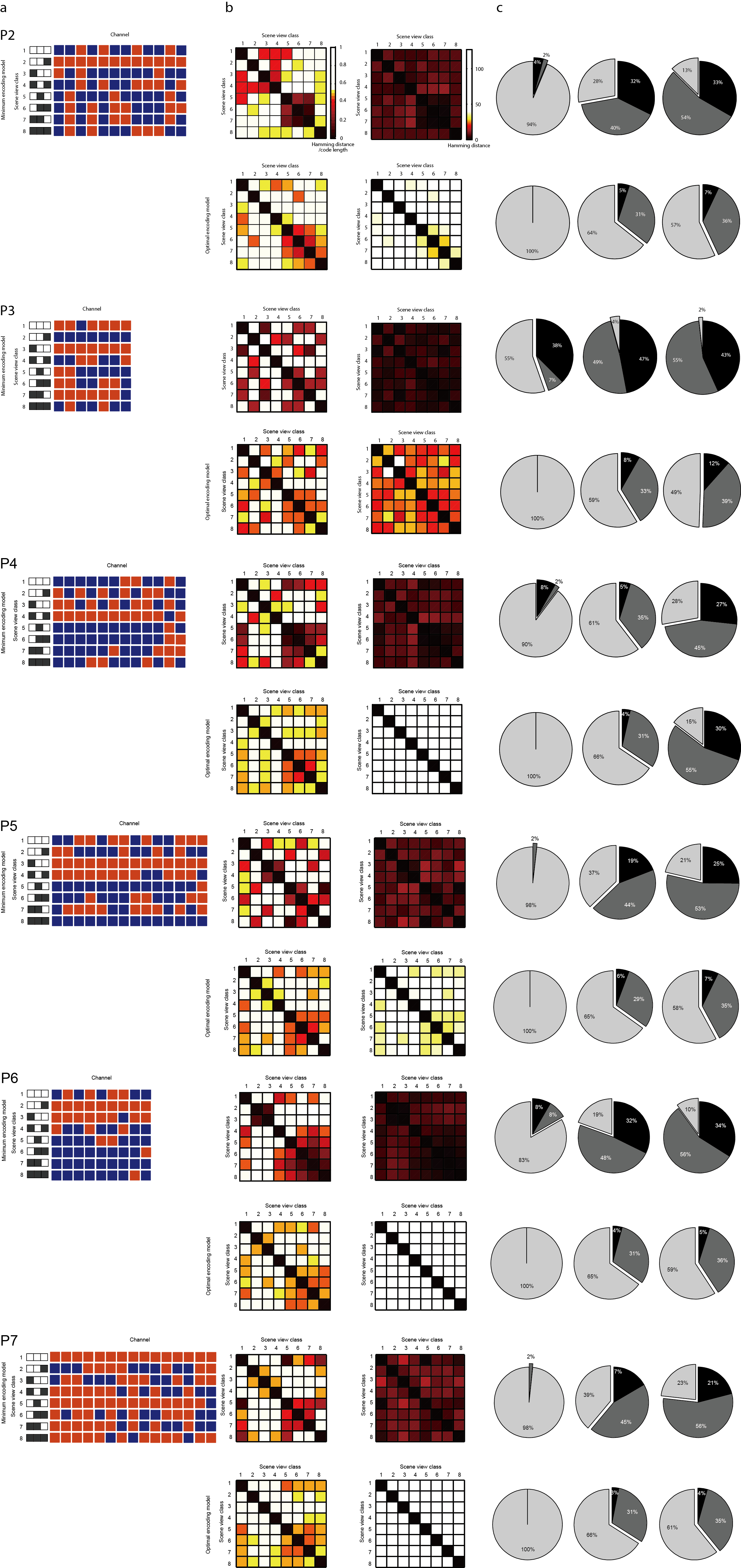
**

**
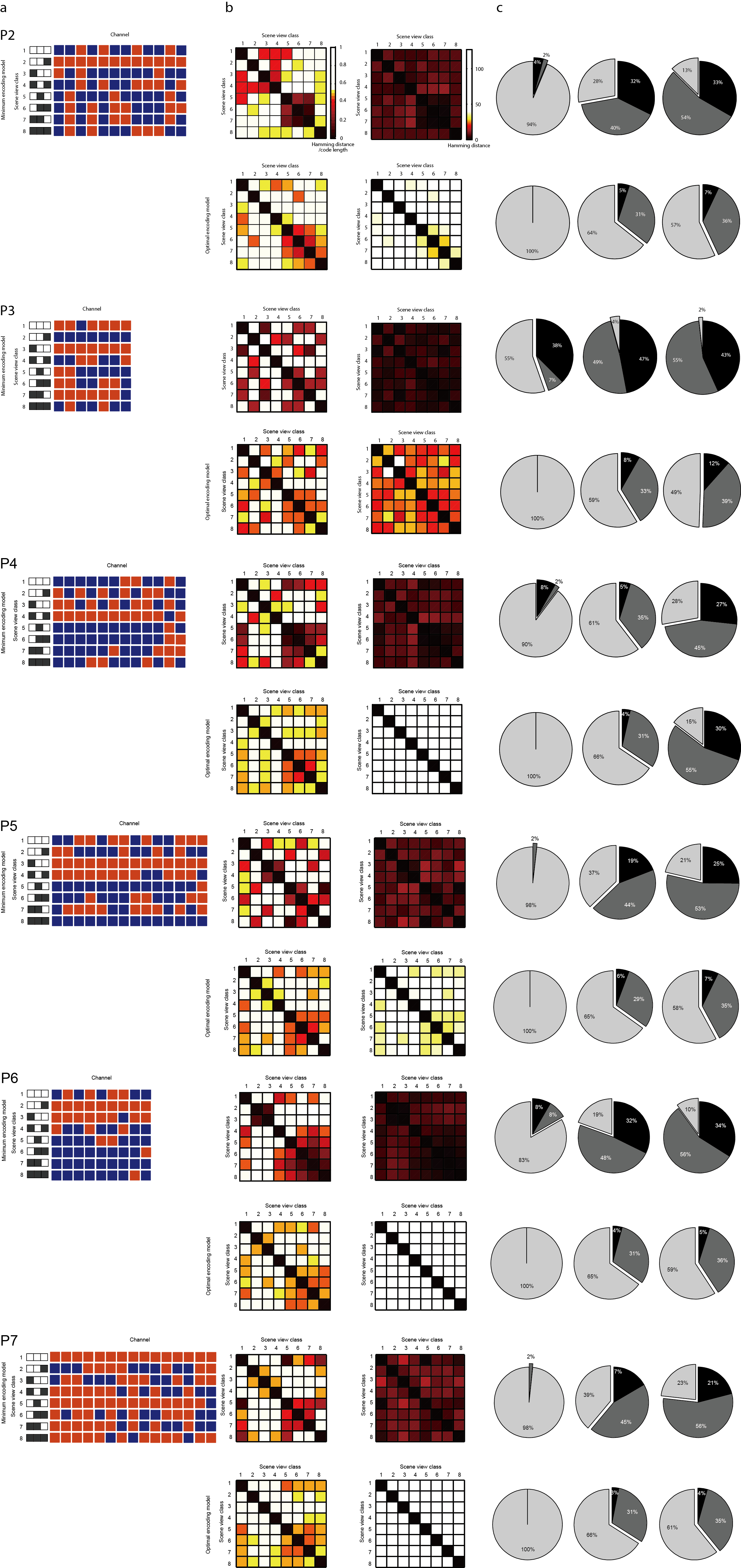
**

Supplementary Figure S2: Minimum encoding model, participants 2-7.

**(a)** The data-driven analysis obtained the minimum and optimal encoding models for each of participants 2-7. Left; the black-white matrix represents the eight types of scene views (white, path; black, wall). Right; the red-blue matrix represents the code matrix (red: positive, ‘1’; blue: negative, ‘0’). Each row, i.e., a code word, in the code matrix corresponds to the scene view on the left. (b) A distance matrix consisting of the Hamming distances between eight scene view classes (top: minimum encoding model, bottom: optimal encoding model). In the left panel, the distance is scaled by the code length, but not in the right panel. (c) A pie chart showing how bit-inversion error will affect Hamming decoding. Light grey, dark grey, and black indicate, respectively, the frequencies with which bit-inversion errors will lead to correct decoding (‘accurate identification’), incorrect decoding (‘misidentification’), and ambiguous cases in which more than two classes become decoding candidates (‘unidentifiable’). Left panels show the case of one bit-inversion error, middle panels the case of two simultaneous bit-inversion errors, and right panels the case of three simultaneous bit-inversion errors.

**
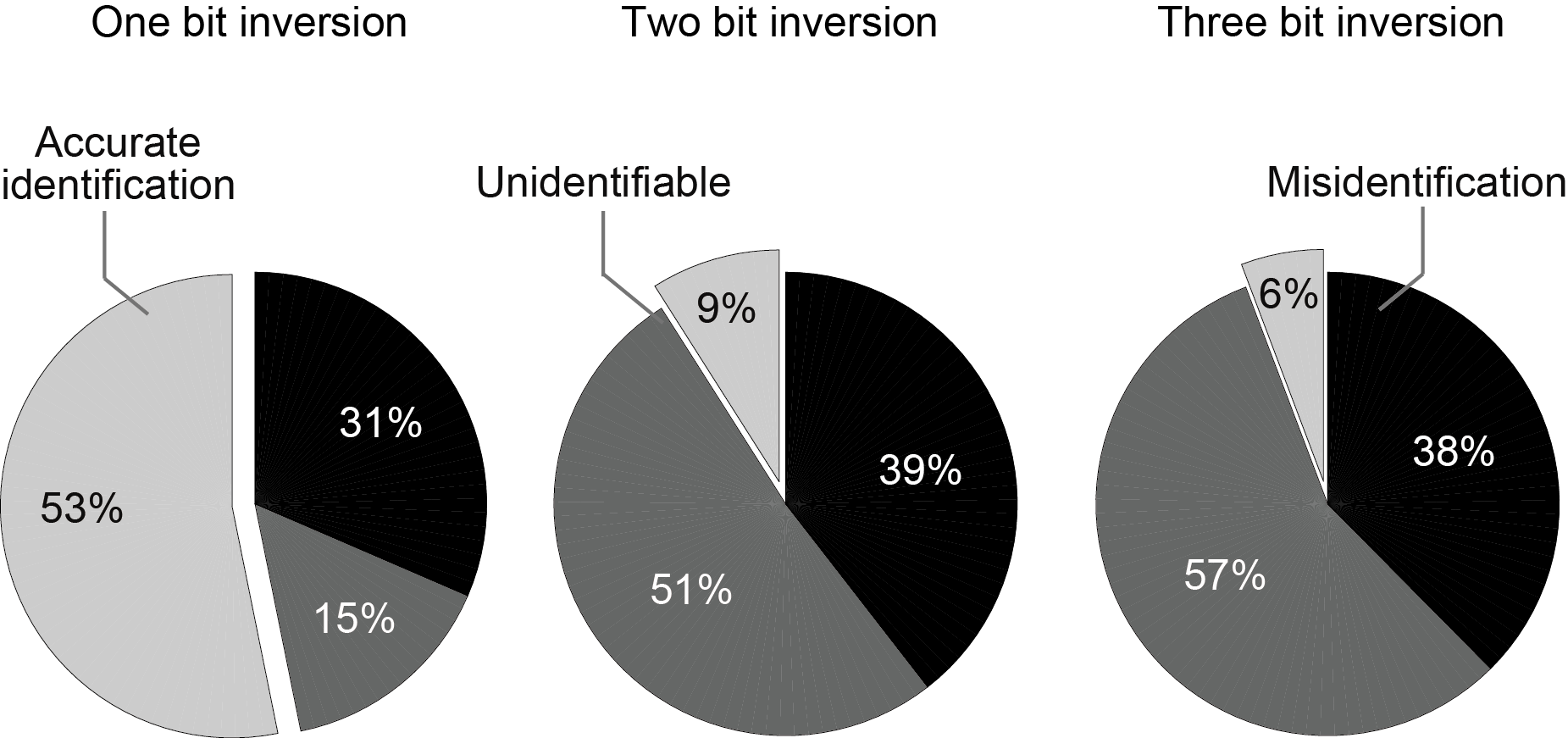
**

Supplementary Figure S3: Effects of bit-inversion errors in the data-unrelated minimum encoding model. Each value was calculated over 1,000 random permutations of the full channel list.


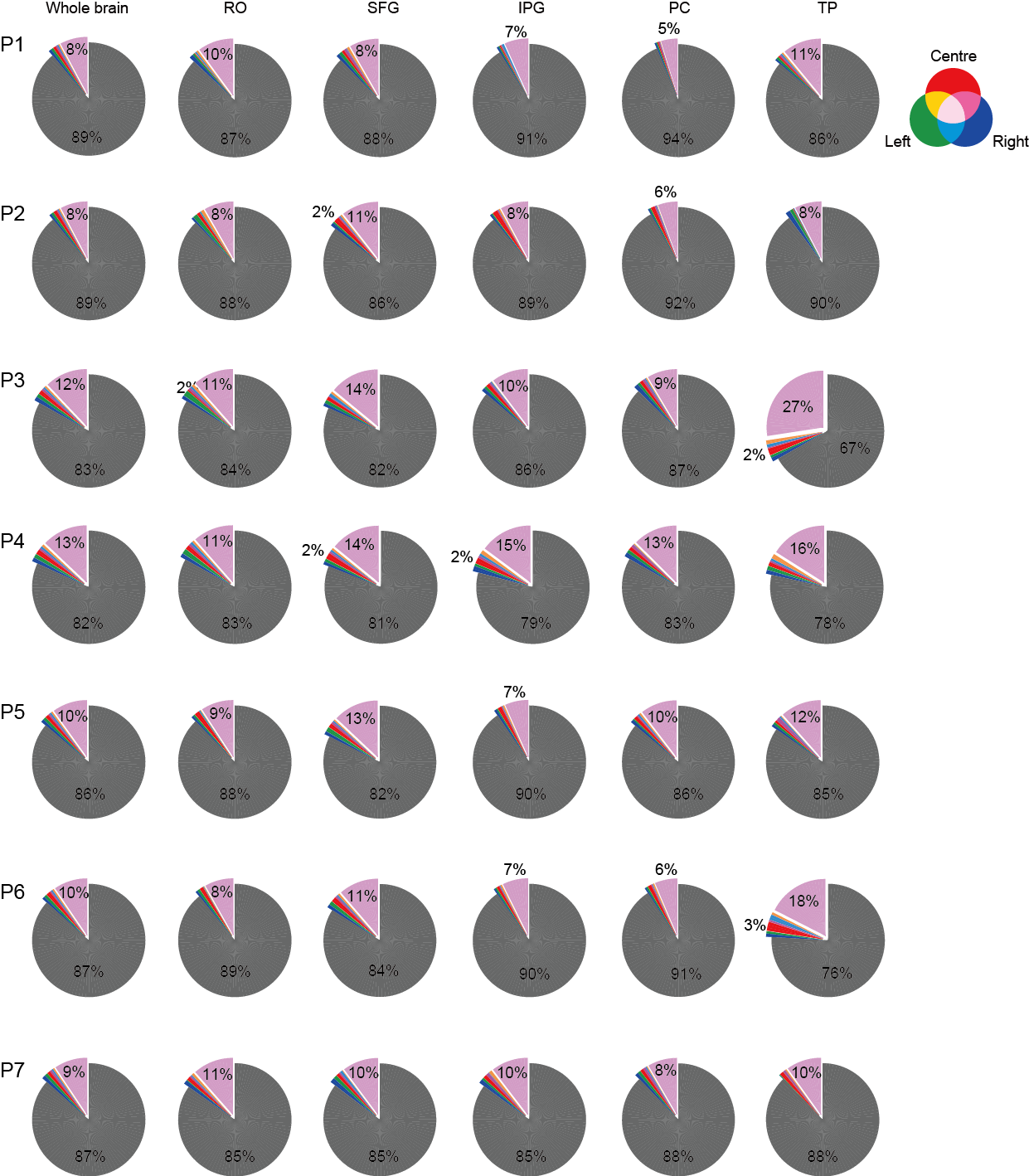


Supplementary Figure S4: Individual results of spatial distributions of the information gain index (IGI). Each row corresponds to a single participant (P1-P7).


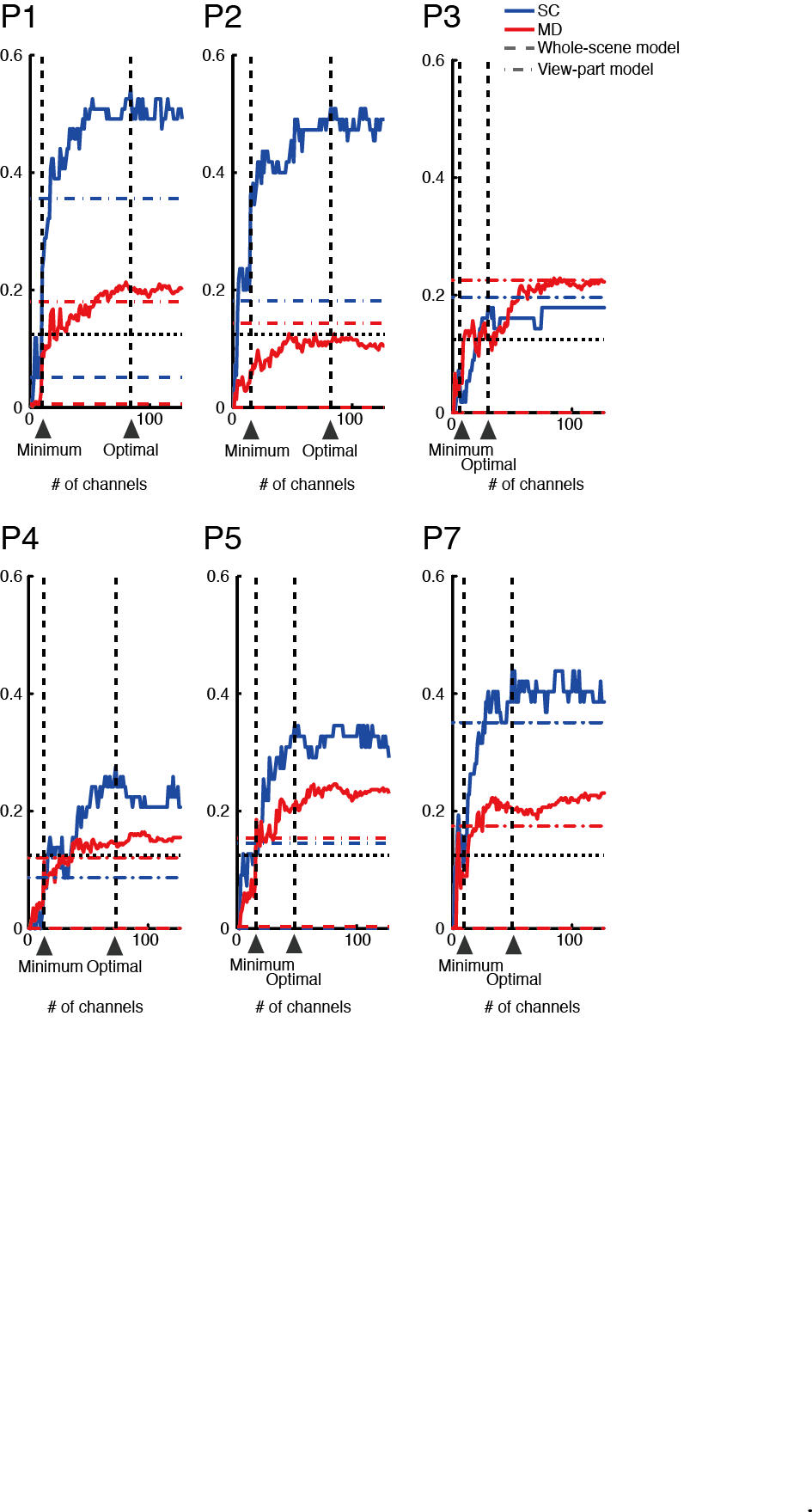


Supplementary Figure S5: Decoding accuracy for participants 2-7. The blue line shows the decoding accuracy validated in the SC sessions (sessions 1, 2, 3, 5, for training, and session 4 for test), and the red line shows the cross-task decoding accuracy for the MD task (SC sessions for training, and the MD session for test). Solid lines represent the number of channels taken from the full encoding list. Dashed lines and dot-and-dash lines indicate decoding accuracies with the whole-scene model and the view-part model, respectively. The meaning of colours is the same as for the solid lines; the red dashed line, i.e., the cross-task decoding accuracy with the whole-scene model, is often close to zero. The horizontal, black dotted line indicates the chance level.
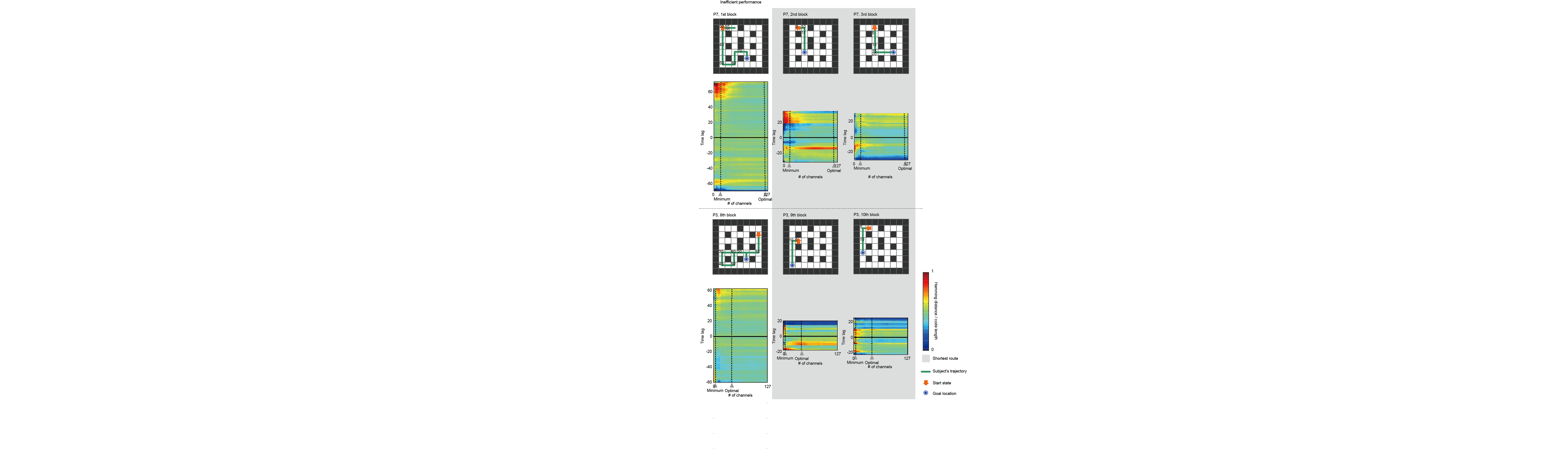


Supplementary Figure S6: Action history and time-shift Hamming distances during the MD task. The time-shift Hamming distance between the code word decoded from current brain activity and one of the correct code words (of the future or past view, being dependent on the positive or negative sign of the time lag, respectively, or the next view when the time lag is zero). Shown are results for three trials, a case where the participant took a detour, resulting in a trial score of 10 or more greater than that of the shortest route (left panel), plus two cases where the participant took the shortest route (middle and right panels). Upper and bottom panels are for participant 7 and participant 3, respectively.
